# Supplementary material for: Ablation of Hepatocyte Derived‐FGL1 Does Not Aggravate Metabolic Dysfunction‐Associated Steatotic Liver Disease
Source: FASEB J. 2025 Aug 20;39(16):e70962. doi: 10.1096/fj.202501997R (PMC12365863; doi:10.1096/fj.202501997R)
Supplement: Supplementary file 1 — Figure S1: fsb270962‐sup‐0001‐Figures.docx. [file FSB2-39-e70962-s001.docx]

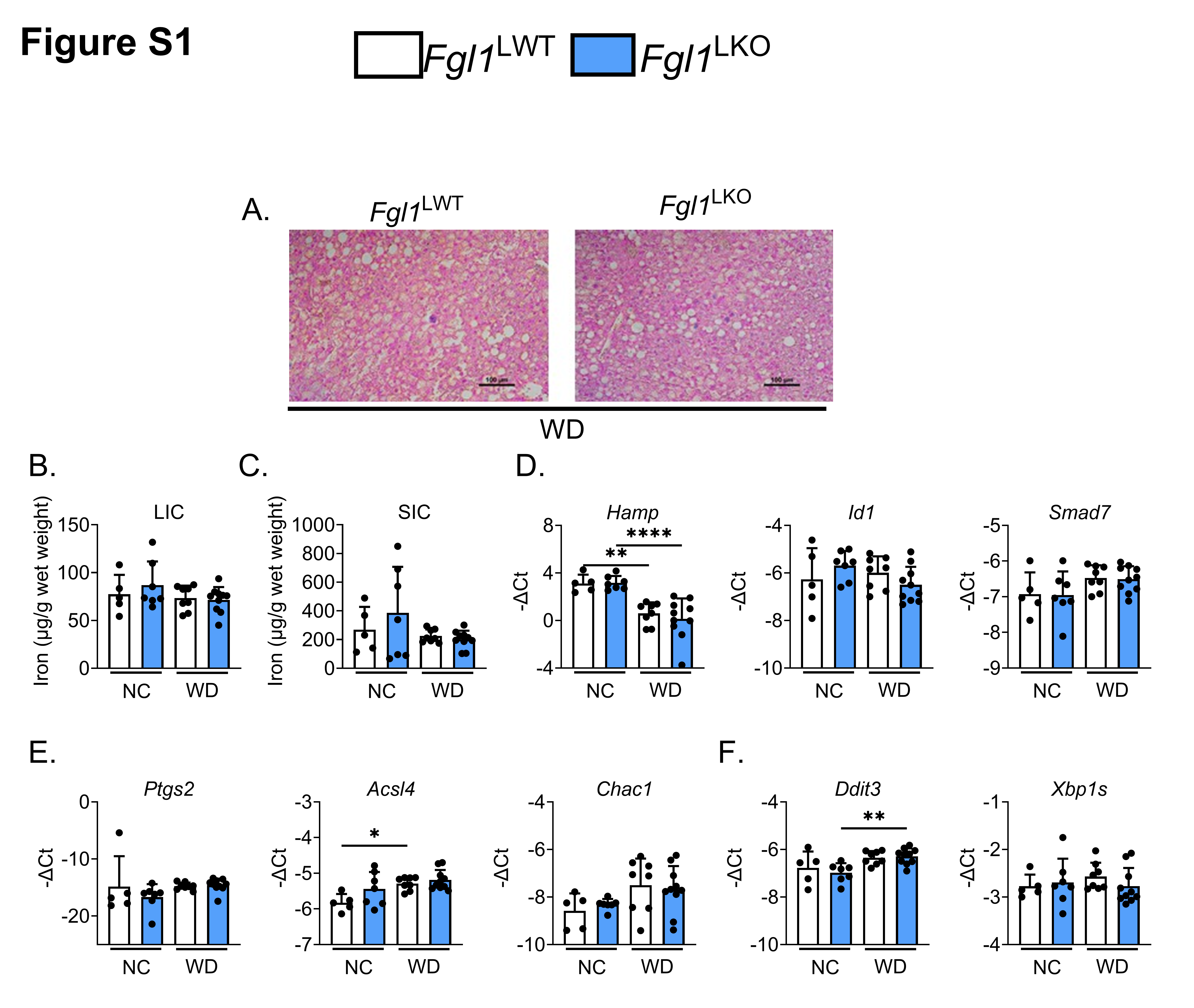


**Figure S1: Deletion of *Fgl1* does not impair iron metabolism during MASLD.** Liver tissue sections were stained with Perls prussian Blue (A). Liver (LIC, B) and spleen (SIC, C) non-heme iron content was measured. Liver mRNA expression of *Hamp, Id1* and *Smad7* (D), *Ptgs2, Acsl4* ,*Chac1* (E), *Ddit3* and *Xbp1s* (F). (n=5-10). Data are means ± SD and were compared by Two-way ANOVA followed by Holm-Šídák's multiple comparisons test, **p<0.01, ***p<0.001, ****p<0.0001.


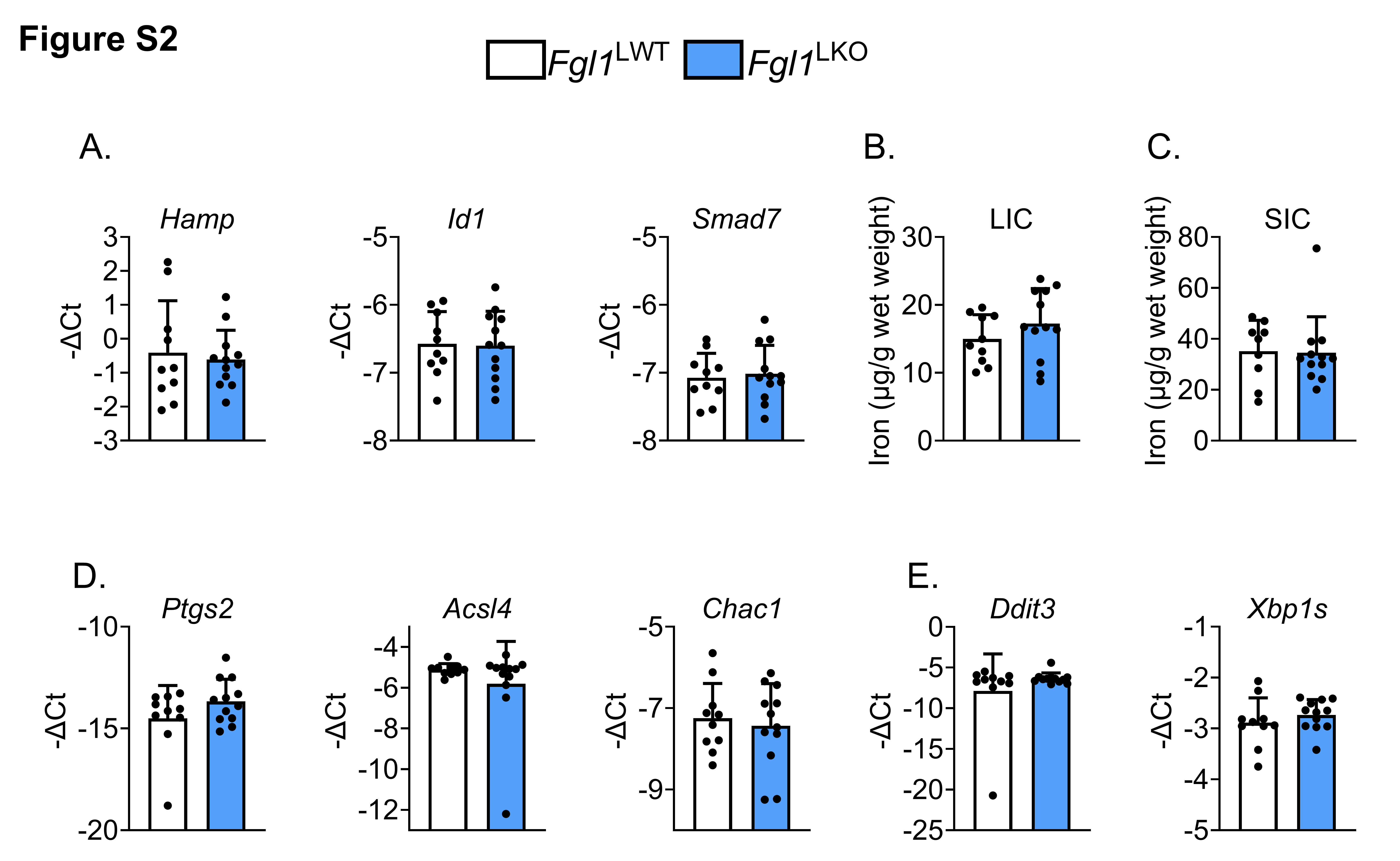


**Figure S2: Deletion of *Fgl1* does not impair iron metabolism during HCC.** Fgl1^LWT^ (n=10) and Fgl1^LKO^ (n=12) male mice were fed a western diet for 24 weeks and given weekly intraperitoneal injections of carbon tetrachloride (CCl4, 0.2µL/g). Liver mRNA expression of *Hamp, Id1* and *Smad7 (A).* Liver (LIC, B) and spleen (SIC, C) non-heme iron content. *Ptgs2, Acsl4* ,*Chac1* (D), *Ddit3* and *Xbp1s* (E) liver mRNA expression. Data are means ± SD and were compared by Student’s t-test.


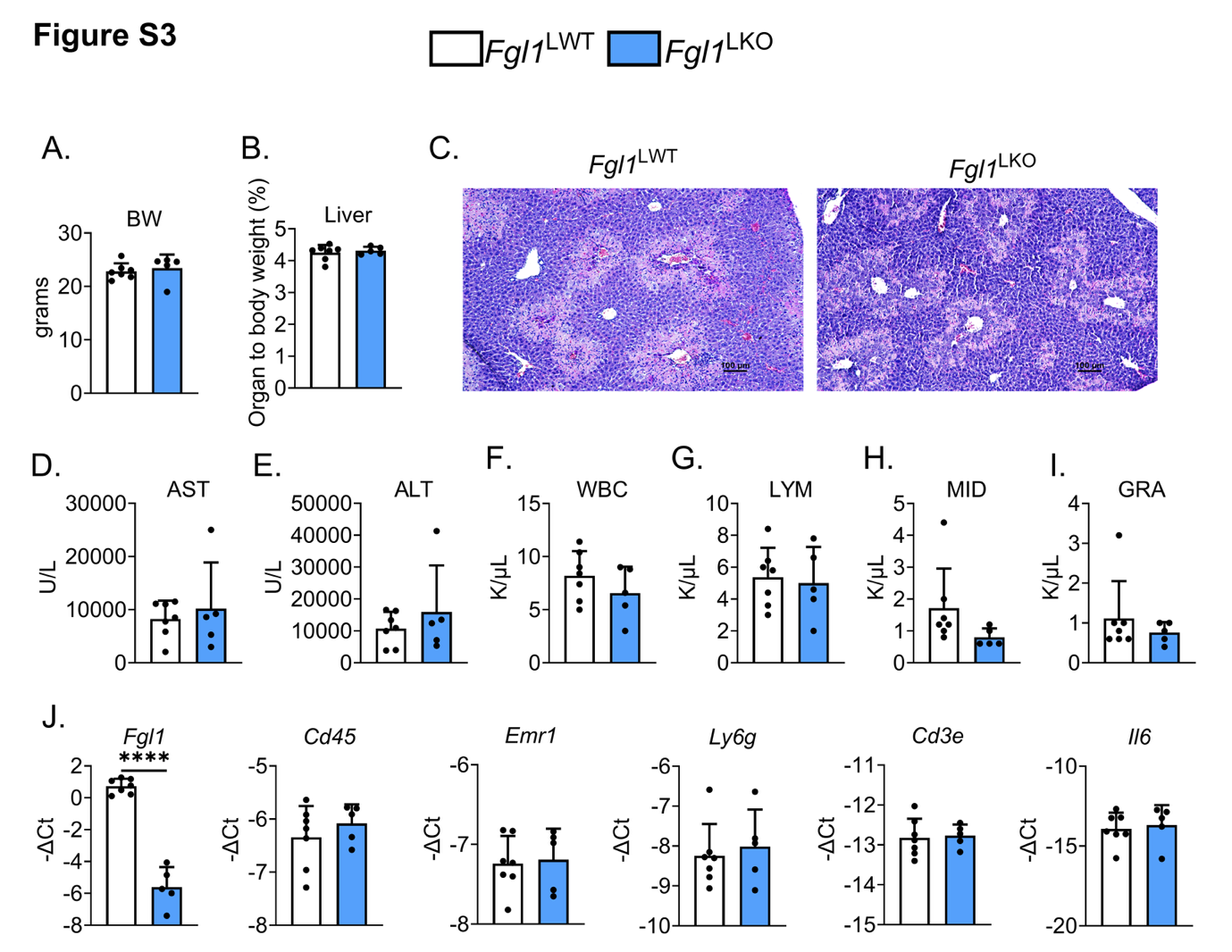


**Figure S3: FGL1 does not influence the inflammatory response to acute liver injury.** *Fgl1* LWT (n=7) and LKO (n=5) male mice were given one intraperitoneal injection of CCl_4_ (0.5µl/g). Body weight (A), liver to body weight ratio (B). Hematoxylin staining of liver tissue sections (C). Circulating alanine (ALT, D) and aspartate (AST, E) aminotransferase levels. Circulating white blood cells (WBC), lymphocytes (LYM), monocytes, eosinophils, basophils, blasts (MID) and neutrophils, monocytes, eosinophils, and basophils (GRA) count. Liver mRNA expression of *Fgl1* (J)*, Cd45, Emr1, Ly6g, Cd3e* and *Il6* (K). Data are means ± SD and were compared by Student’s t-test. ****p<0.0001.
